# First instar and adult male bed bugs, *Cimex lectularius* (Hemiptera: Cimicidae), increase feeding activity in the presence of adult females

**DOI:** 10.1186/s13071-024-06289-3

**Published:** 2024-07-08

**Authors:** Sydney E. Crawley, John H. Borden, Josiah P. Ritchey, Kenneth F. Haynes

**Affiliations:** 1 Department of Entomology, College of Agriculture, Food and Environment, University of Kentucky, S-225 Agricultural Science Center, Lexington, KY 40546 USA; 2 JHB Consulting, 6552 Carnegie Street, Burnaby, BC V5B 1Y3 Canada; 3 Department of Entomology and Plant Pathology, North Carolina State University, 100 Derieux Place, Campus Box 7613, Raleigh, NC 27695-7613 USA; 4 Present Address: Rentokil-Terminix, 9000 Freeport Parkway, Suite 100, Irving, TX 75063 USA

**Keywords:** Bed bug, Cimicidae, Feeding, First instar

## Abstract

**Background:**

Bed bugs, *Cimex lectularius*, form day-time aggregations from which they depart at night to feed on human blood. Obtaining an initial blood meal is a critical step in the development of first instars. Previous research had shown that first instars had greater success in obtaining this essential meal when in the presence of adults than when they were alone.

**Methods:**

Feeding by bed bugs was tested in upright vertical cylindrical chambers fitted with a paper ramp to aid in climbing toward a blood feeder suspended across the upper end of the cylinder. Feeding success by the first instars was tested when they were alone in the chamber or when they were in the presence of adult females, males, or both together.

**Results:**

The mean proportions of the first instars that fed were significantly higher when they were confined with adults of both sexes or adult females than when they were confined alone or with males. Feeding by adult males was also enhanced by confinement with females. When first instars and adult females were confined together, the mean duration before first instars began feeding was longer than for females. There was no difference in feeding success by first instars confined with their mothers or nonmothers.

**Conclusions:**

Elevated feeding by first instars and adult males in the presence of females may be adaptive traits that enhance fitness. First instars must feed to avoid dehydration and starvation and to obtain resources needed for development. Adult males would benefit not only by increased feeding success but also by greater likelihood of finding a recently engorged female with which to mate. The lack of any difference in feeding success of first instars in the presence of their mothers or nonmothers argues against parental care in this species.

**Graphical Abstract:**

**Figure:**
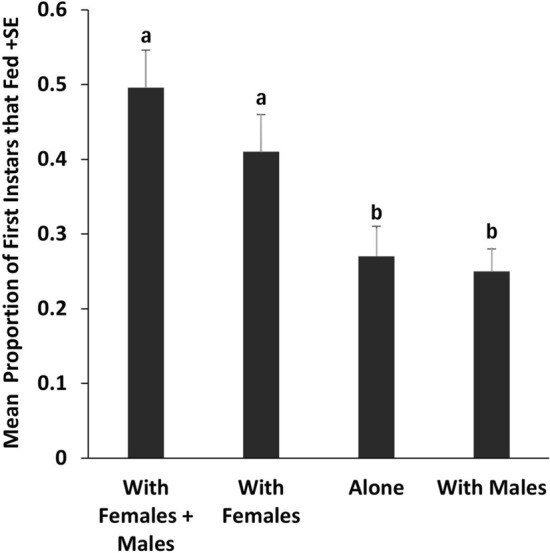

**Supplementary Information:**

The online version contains supplementary material available at 10.1186/s13071-024-06289-3.

## Background

Many nonsocial animals benefit from aggregation through defense against predators, microhabitat modification, sharing of symbionts, improved mate-finding, increased foraging efficiency, and parental care [1, 2]. Conversely, the costs of aggregation include proximity-induced disease transmission, cannibalism, and competition for scarce resources [3]. Regarding foraging, if the benefits of aggregation are high, then selection should favor communication mechanisms that complement resource-associated cues. Such cues are common and span every sensory modality across various species [3].

Bed bugs, *Cimex lecturarius* (Hemiptera: Cimicidae), are blood-feeding ectoparasites [4, 5] that form large, pheromone-mediated aggregations [6] that they leave at night to feed on sleeping hosts [7]. The proximity of a stationary host improves the likelihood of obtaining the five blood meals needed to reach adulthood and the additional meals needed to maximize life-time reproduction [5, 8]. Within an aggregation, optimized humidity and/or temperature may foster development of immatures [9–11].

The presence of fed adult bed bugs of mixed sex within an aggregation has been shown to slightly improve feeding success of first instars [12]. We expand on this finding by testing the hypothesis that feeding by first instars will be enhanced in the presence of either sex of adult bed bugs.

## Methods

### Experimental insects

Bed bugs were obtained from a colony established in 2008 with approximately 100 bed bugs collected from Plainview, NY. The colony was maintained at 27 °C, 70% relative humidity, and 14:10 light:dark (L:D) and fed weekly on defibrinated rabbit blood (Quad Five, Rygate, MT) pipetted into glass feeders (Kimble Chase Custom Glass Shop, Vineland, NJ) and heated to 39 °C with a circulating water bath [13]. To feed, bed bugs contained in 59 mL plastic jars (Consolidated Plastics, Stow, OH) projected their proboscis through a layer of organza covering the open end of the jar and pierced a parafilm membrane lining the bottom of the feeder. Adults were used in experiments 6–7 days after feeding. The first instars were of mixed age 1–5 days old and unfed after hatching. Within a replicate, all bugs were from the same jar.

### Experiments   

In Experiment 1, bed bugs were placed in vertical cylindrical glass chambers, 25 cm high × 10 cm diameter, covered with organza secured by a rubber band. A 2.5-cm-wide strip of paper glued to the inside wall as a “ramp” allowed the bed bugs to climb to the top. A 4 cm × 4 cm piece of blotting paper leaning against the wall provided a harborage at the bottom.

For each of 20 replicates, randomized treatments included 30 first instars alone or with 10 adult males, 10 adult females, or with 5 males and 5 females. The bed bugs were held at 14:10 L:D in cylinders for 24 h before testing. For each replicate, four cylinders were placed on a level table and feeders were placed over the organza ceilings. A bed bug would walk up the ramp and feed through the organza and parafilm as above. To enhance the host-related stimuli associated with a blood meal, a plastic tube (1.6 mm internal diameter) delivered air enriched with 5% CO_2_ through the organza at 1 L/min into each cylinder on a 30/15-min on/off cycle. The bioassays were conducted under red light so as not to disturb bed bugs during observations, which began approximately 3 h into the scotophase and lasted 4 h. Afterward, the numbers of engorged and nonengorged bugs were recorded.

Experiment 2 compared the time until feeding by females and first instars when they were confined together. Cylindrical feeding chambers, 12 cm high × 10 cm diameter with a 12 cm × 3 cm paper ramp, were mounted vertically above the lens of an infrared-sensitive digital camera (Sony CyberShot, San Diego, CA). Fifteen females and 15 first instars were placed into each of eight cylinders (replicates). Ten min later, a stream of 5% CO_2_ in air was directed into each cylinder. The bugs were allowed to blood-feed as above for 1 h beginning at 3 h into the scotophase. Photographs taken every 30 s by programmed remote trigger (Palm m125, Palm, Inc., Sunnyvale, CA) with OmniRemote Software (Lutron Electronics Co., Inc., Coopersburg, PA) were examined to determine initiation of feeding.

Experiment 3 compared the effect of biological mothers against “foster” mothers on first instar feeding. Climbing was facilitated by replacing the vertical ramp with a 25 cm × 2.5 cm strip of stiff blotting paper with filter paper affixed to both sides with double sided sticky tape. This structure stood free with only the top touching the wall and organza. Based on repeated observations that behavioral outcomes were not impacted, the acclimation period was reduced to 5 min and CO_2_ was not provided.

To obtain related offspring and parents for Experiment 3, individual adult males and females were placed in 59 mL sealed polyethylene cups directly after feeding and allowed 24 h to mate. The females were then held individually for 10 days in 15-cm-diameter petri dishes lined with filter paper for egg laying [13]. After all eggs hatched, five first instars were removed and placed in 25 cm × 10 cm diameter cylinders. Individual females were randomly placed in ten cylinders containing their own offspring or in ten cylinders with “foster” nymphs. Feeding by females and first instars was permitted for 60 min.

### Statistical analyses

Statistix 10.0 (Analytical Software, Tallahassee, FL) and R version 4.0.3 were used for analyses. For each replicate in Experiment 1, the proportions of first instars that fed were arcsine square-root transformed prior to analysis to impart normalcy [14], which was confirmed using a Shapiro–Wilk test. Transformed proportions of first instars that fed in Experiment 1 were analyzed by fitting a linear mixed-effect model (*LME4* package) [15] with treatment as a fixed effect and replicate (*N* = 20) as a random effect. Planned linear contrasts (*EMMEANS* package) [16] were used to evaluate differences in the transformed proportions of first instars that fed among treatments. *t*-Tests were used to compare untransformed proportions of adult males or females feeding in Experiment 1, mean durations before initiation of feeding in Experiment 2, and untransformed proportions of fed first instars confined with their mothers or nonmothers in Experiment 3. In all cases, *α* = 0.05.

## Results

**Fig. 1:**
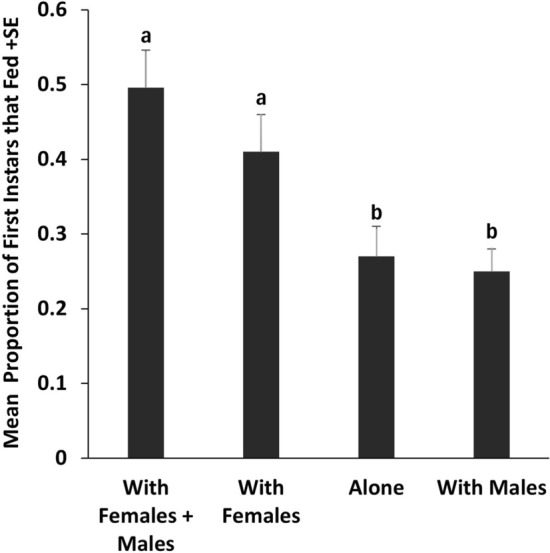
Mean proportions in Experiment 1 of first instar bed bugs (30 per replicate, *N* = 20) that fed in upright cylindrical feeding chambers also containing adult females plus adult males, adult females, no adults, or adult males. Bars with the same letters are not significantly different (linear mixed-effect model, *P* < 0.05).

In Experiment 1, first instars fed with significantly different levels of success depending on the treatment (*F*_3,57_ = 8.797, *P* = 0.0000694) (Fig. 1). Adult females had a significant positive effect on the proportions of first instars that fed. Compared with feeding in the absence of adults, the mean proportion of first instars feeding was 79% higher in the presence of adults of both sexes (*t*_57_ = 4.06, *P* < 0.001) and 46% higher in the presence of adult females (*t*_57_ = 2.678, *P* = 0.0463). There was no effect of the presence of adult males on the proportion of first instars feeding (*t*_57_ = *−0.25*, *P* = 0.995).

**Fig. 2:**
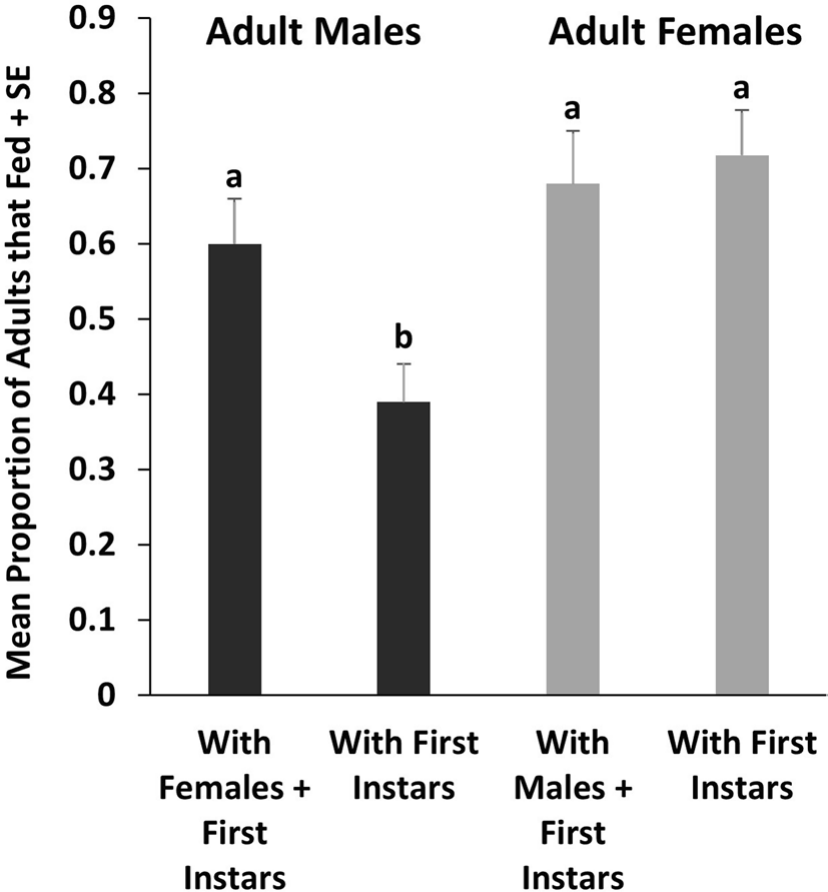
Mean proportions in Experiment 1 of adult males (black bars) that fed in the presence of adult females plus first instars or first instars alone and adult females (gray bars) that fed in the presence of adult males plus first instars or first instars alone. For each sex, bars with the same letters are not significantly different (*t*-test, *P* < 0.05).

The mean proportion of adult males that fed in the presence of adult females in Experiment 1 was significantly higher (by 54%) than when males were in the feeding chamber with first instars (*t*_38_ = 2.5028, *P* = 0.0157) (Fig. 2). Females fed in nearly equal proportions whether or not males were present (*t*_38_ = 0.3896, *P* = 0.6990) (Fig. 2).

**Fig. 3:**
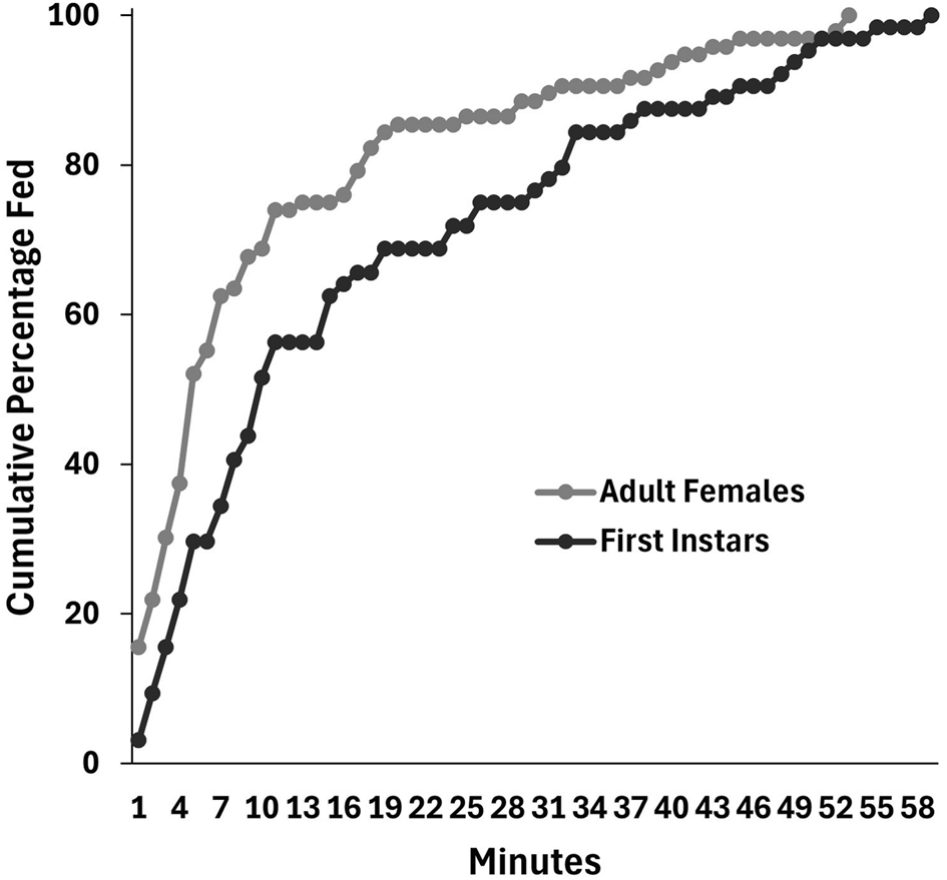
Cumulative percentages of 96 adult female and 64 first instar bed bugs in Experiment 2 initiating feeding over time in upright cylindrical feeding chambers. Female bed bugs initiated feeding significantly more quickly than first instars (*t*-test, *P* < 0.05).

In Experiment 2, 80.0% of 120 adult females and 53% of 120 first instars fed. Fifteen adult females and two first instars began feeding within 1 min after provision of blood (Fig. 3). Final initiation of feeding by a female occurred in the fifty-third minute, while the last initiation of feeding by a first instar occurred in the fifty-ninth minute. Excluding nonfeeders, the mean durations before females and first instars began to feed were 11.10 ± 1.34 min and 17.42 ± 2.00 min, respectively (*t*_158_ = 2.7342, *P* = 0.007).

**Fig. 4:**
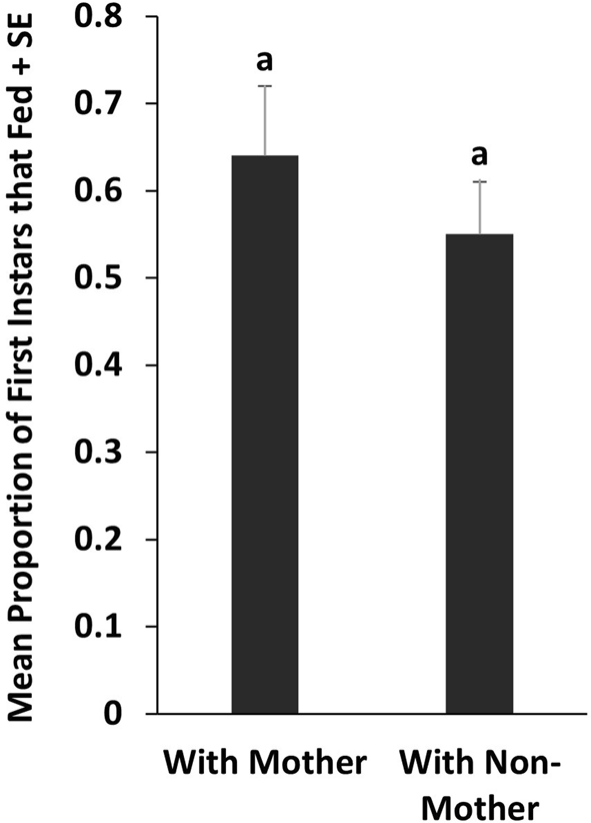
Mean proportions of first instar bed bugs in Experiment 3 that fed in upright cylindrical feeding chambers in the presence of adult females that were either their mother or a nonmother. Bars with the same letters are not significantly different (*t*-test, *P* < 0.05).

In Experiment 3, there was no difference in the proportion of first instars that fed when in feeding chambers with their mother (64%) or a non-mother (55%) (*t*_38_ = 0.9517, *P* = 0.3472) (Fig. 4).

## Discussion

Our results, as well as preliminary findings by Crawley et al. [17], demonstrate that the presence of adult female (but not male) bed bugs increases feeding by first instars (Fig. 1). In addition, feeding by adult males was significantly higher when they were in the same feeding chamber as females and first instars than when they were with first instars alone (Fig. 2), suggesting an additional effect of females on male feeding.

Balvín et al. [12] reported that in the presence of fed adult bed bugs of both sexes, feeding success of first instars was enhanced by approximately 20% (our interpretation of their Fig. 1), much lower than the effects we found for first instars in the presence of females (Fig. 1). They described the enhancement phenomenon as “adults helping nymphs to locate a food source.” We propose a different hypothesis, in which first instars and adult males exploit a female-emitted cue that enhances fitness by increasing the likelihood of obtaining a blood meal. First instars are particularly susceptible to dehydration and starvation [10]. Males must find and mate with a recently engorged female to ensure optimal siring of offspring [18, 19]. Thus, it would benefit first instars to exploit female-produced cues for the purposes of feeding and would benefit adult males for the purpose of feeding plus mating. The latter hypothesis should be explored further in experiments designed to test the effect of each adult sex on the other in the absence of nymphs.

Balvin et al. [12] apparently established 16 colonies, each with five female and two male adult bed bugs. After 1 week, the adults were removed from half of the colonies, leaving the eggs and newly hatched first instars. Feeding for 30 min was initiated after 2 weeks and apparently was done weekly for the next 2 weeks for 48 total measurements. For all 16 colonies, there was a significant positive relationship between the total number of adults present and the proportion of nymphs that fed. For the eight colonies with the adults left in place, there were significant positive relationships between both the proportion of fed adults and the number of nymphs present and the proportion of nymphs that fed. Curiously, there was a negative relationship between the total number of adults present and the proportion of nymphs that fed. Although our results support those of Balvin et al. [12] in part and assign the positive stimulatory effect to adult females (Fig. 1), they are not directly comparable because we did not make multiple evaluations of the same groups of bed bugs over time.

On average, first instars lagged behind females in beginning to feed by approximately 6 min (Fig. 3), and some females were observed returning to harborages before the first instars attempted to feed. Both observations suggest that first instars delayed initiation of feeding until a female-produced cue was produced during or shortly after feeding. This result plus the finding in Experiment 1 of enhanced feeding by first instars in the presence of adult females suggests that in rearing, immature bed bugs should not be separated from adults until they have at least reached the second instar.

Because engorged males failed to induce enhanced feeding by first instars, chemical or thermal cues associated with ingested warm blood are unlikely, supporting the hypothesis that the stimulus for enhanced feeding by first instars and adult males is female produced. The unknown female-produced cue must be more specific than microbe-produced kairomones or nymph-produced pheromones that, respectively, induce feeding aggregations in flies (Diptera) [20, 21] and other hemipterans [3]. It must also be more specific than the bed bug aggregation pheromone, which is produced by and mediates the behavior of both sexes and all life stages [6]. One possible chemical cue is eucalyptol, the predominant volatile associated with bed bug eggs [22], which could be deposited by females soon after feeding, as well as during apparent egg marking behavior [23]. Another hemipteran, the cotton seed bug, *Oxycarenus hyalinipennis*, (Hemiptera: Lygaeidae) produces eucalyptol in the metathoracic scent glands [24]. However, the origin and functionality of eucalyptol in bed bugs has yet to be determined.

B﻿alvin et al. [12] suggest that their finding of enhanced feeding by first instars in the presence of fed adults supports the classification of bed bugs as subsocial. Our results do not support this hypothesis. In Experiment 3, first instars confined in feeding chambers with their mothers initiated feeding at the same levels as when they were confined with nonmothers (Fig. 3), indicating that females do not preferentially communicate with their own offspring, a phenomenon that would have been indicative of parental care and would have supported classification of bed bugs as subsocial [1, 25].

## Conclusion

We have shown that the presence of adult female C. lectularius stimulates first instars and adult males to feed on blood at significantly higher levels than when females are absent and that first instars lag behind adult females in initiating feeding when they are confined together. These results suggest that first instars and adult males increase their fitness by exploiting a female-produced stimulatory cue that enhances the likelihood of obtaining a critical blood meal. The finding of similar feeding by first instars when confined with their mothers or non-mothers argues against parental care in this species.

### Supplementary Information

**Additional file 1: Table S1.** Data from Experiment 1 used in compiling Fig. 1. Proportions of 30 first instar *Cimex lectularius* that fed in four treatments.**Table S2.** Data from Experiment 1 used in compiling Fig. 2. Proportions of adult male and adult female *Cimex lectularius* that fed when confined with first instars alone or with an adult of the opposite sex. *Table S3.* Data from Experiment 2 used in compiling Fig. 3. Cumulative percentages of adult female and first instar *Cimex lectularius* that fed when confined in the same chamber. **Table S4.** Data from Experiment 3 used in compiling Fig. 4. Proportions of five first instar *Cimex lectularius* that fed when confined with their mother or a non-mother.

## Data Availability

The data for Figs. 1-4 are provided in Additional files 1: Tables S1, S2, S3 and S4.
