# Supplementary material for: First instar and adult male bed bugs, Cimex lectularius (Hemiptera: Cimicidae), increase feeding activity in the presence of adult females
Source: Parasit Vectors. 2024 Jul 8;17:293. doi: 10.1186/s13071-024-06289-3 (PMC11232164; doi:10.1186/s13071-024-06289-3)
Supplement: Supplementary file 1 — Additional file 1: Table S1. Data from Experiment 1 used in compiling Fig. 1. Proportions of 30 first instar Cimex lectularius that fed in four treatments.Table S2. Data from Experiment 1 used in compiling Fig. 2. Proportions of adult male and adult female Cimex lectularius that fed when confined with first instars alone or with an adult of the opposite sex. Table S3. Data from Experiment 2 used in compiling Fig. 3. Cumulative percentages of adult female and first instar Cimex lectularius that fed when confined in the same chamber. Table S4. Data from Experiment 3 used in compiling Fig. 4. Proportions of five first instar Cimex lectularius that fed when confined with their mother or a non-mother. [file 13071_2024_6289_MOESM1_ESM.docx]

**Additional file 1: Table S1.** Data from Experiment 1 used in compiling Fig. 1. Proportions of 30 first instar *Cimex lectularius* that fed in four treatments.

| **Replicate** | **Proportion of first instars that fed** | | | |
| --- | --- | --- | --- | --- |
|  | **With adult females + males** | **With adult females** | **Alone** | **With adult males** |
| 1 | 0.38 | 0.76 | 0.21 | 0.33 |
| 2 | 0.40 | 0.48 | 0.26 | 0.34 |
| 3 | 0.33 | 0.03 | 0.20 | 0.07 |
| 4 | 0.17 | 0.23 | 0.10 | 0.50 |
| 5 | 0.67 | 0.19 | 0.46 | 0.11 |
| 6 | 0.19 | 0.59 | 0.36 | 0.25 |
| 7 | 0.37 | 0.18 | 0.37 | 0.17 |
| 8 | 0.67 | 0.87 | 0.47 | 0.04 |
| 9 | 0.81 | 0.50 | 0.30 | 0.52 |
| 10 | 0.17 | 0.33 | 0.57 | 0.53 |
| 11 | 0.77 | 0.39 | 0.25 | 0.19 |
| 12 | 0.77 | 0.65 | 0.31 | 0.32 |
| 13 | 0.60 | 0.50 | 0.00 | 0.17 |
| 14 | 0.54 | 0.40 | 0.32 | 0.27 |
| 15 | 0.57 | 0.52 | 0.31 | 0.30 |
| 16 | 0.64 | 0.60 | 0.35 | 0.30 |
| 17 | 0.67 | 0.52 | 0.50 | 0.19 |
| 18 | 0.37 | 0.20 | 0.08 | 0.20 |
| 19 | 0.33 | 0.20 | 0.08 | 0.20 |
| 20 | 0.50 | 0.23 | 0.07 | 0.10 |

**Additional file 1: Table S2.** Data from Experiment 1 used in compiling Fig. 2. Proportions of adult male and adult female *Cimex lectularius* that fed when confined with first instars alone or with an adult of the opposite sex.

| **Replicate** | **Proportion of adult males that fed** | | **Proportion of adult females that fed** | |
| --- | --- | --- | --- | --- |
|  | **With females + first instars** | **With first instars** | **With males + first instars** | **With first instars** |
| 1 | 0.80 | 0.40 | 0.80 | 0.70 |
| 2 | 0.40 | 0.50 | 0.20 | 0.70 |
| 3 | 0.40 | 0.60 | 0.80 | 0.80 |
| 4 | 0.60 | 0.50 | 0.80 | 0.90 |
| 5 | 0.80 | 0.20 | 0.80 | 0.70 |
| 6 | 0.40 | 0.50 | 0.00 | 0.80 |
| 7 | 0.80 | 0.40 | 0.80 | 0.10 |
| 8 | 0.20 | 0.30 | 0.40 | 1.00 |
| 9 | 0.00 | 0.70 | 0.80 | 0.70 |
| 10 | 0.40 | 0.50 | 0.20 | 0.60 |
| 11 | 0.80 | 0.56 | 1.00 | 0.70 |
| 12 | 0.60 | 0.10 | 1.00 | 1.00 |
| 13 | 0.20 | 0.10 | 0.60 | 0.78 |
| 14 | 1.00 | 0.80 | 0.80 | 0.90 |
| 15 | 0.80 | 0.00 | 1.00 | 1.00 |
| 16 | 0.75 | 0.30 | 0.60 | 1.00 |
| 17 | 0.60 | 0.30 | 0.80 | 0.60 |
| 18 | 1.00 | 0.30 | 0.80 | 0.30 |
| 19 | 0.40 | 0.10 | 0.40 | 0.20 |
| 20 | 1.00 | 0.70 | 1.00 | 0.80 |

**Additional file 1: Table S3.** Data from Experiment 2 used in compiling Fig. 3. Cumulative percentages of adult female and first instar *Cimex lectularius* that fed when confined in the same chamber.

| **Minute** | **Cumulative percentage of adult females fed (N = 96)** | **Cumulative percentage of first instars fed (N = 53)** |
| --- | --- | --- |
| 1 | 15.6 | 3.1 |
| 2 | 21.9 | 9.4 |
| 3 | 30.2 | 15.6 |
| 4 | 37.5 | 21.9 |
| 5 | 52.1 | 29.7 |
| 6 | 55.2 | 29.7 |
| 7 | 62.5 | 34.4 |
| 8 | 63.5 | 40.6 |
| 9 | 67.7 | 43.8 |
| 10 | 68.8 | 51.6 |
| 11 | 74 | 56.3 |
| 12 | 74 | 56.3 |
| 13 | 75 | 56.3 |
| 14 | 75 | 56.3 |
| 15 | 75 | 62.5 |
| 16 | 76 | 64.1 |
| 17 | 79.2 | 65.6 |
| 18 | 82.3 | 65.6 |
| 19 | 84.4 | 68.8 |
| 20 | 85.4 | 68.8 |
| 21 | 85.4 | 68.8 |
| 22 | 85.4 | 68.8 |
| 23 | 85.4 | 68.8 |
| 24 | 85.4 | 71.9 |
| 25 | 86.5 | 71.9 |
| 26 | 86.5 | 75 |
| 27 | 86.5 | 75 |
| 28 | 86.5 | 75 |
| 29 | 88.5 | 75 |
| 30 | 88.5 | 76.6 |
| 31 | 89.6 | 78.1 |
| 32 | 90.6 | 79.7 |
| 33 | 90.6 | 84.4 |
| 34 | 90.6 | 84.4 |
| 35 | 90.6 | 84.4 |
| 36 | 90.6 | 84.4 |
| 37 | 91.7 | 85.9 |
| 38 | 91.7 | 87.5 |
| 39 | 92.7 | 87.5 |
| 40 | 93.8 | 87.5 |
| 41 | 94.8 | 87.5 |
| 42 | 94.8 | 87.5 |
| 43 | 95.8 | 89.1 |
| 44 | 95.8 | 89.1 |
| 45 | 96.9 | 90.6 |
| 46 | 96.9 | 90.6 |
| 47 | 96.9 | 90.6 |
| 48 | 96.9 | 92.2 |
| 49 | 96.9 | 93.8 |
| 50 | 96.9 | 95.3 |
| 51 | 96.9 | 96.9 |
| 52 | 97.9 | 96.9 |
| 53 | 100 | 96.9 |
| 54 |  | 96.9 |
| 55 |  | 98.4 |
| 56 |  | 98.4 |
| 57 |  | 98.4 |
| 58 |  | 98.4 |
| 59 |  | 100 |

**Additional file 1: Table S4.** Data from Experiment 3 used in compiling Fig. 4. Proportions of five first instar *Cimex lectularius* that fed when confined with their mother or a non-mother.

|  | **Proportion of first instars that fed** | |
| --- | --- | --- |
| **Replicate** | **With mother** | **With non-mother** |
| 1 | 0.8 | 0.4 |
| 2 | 1 | 0.4 |
| 3 | 0.8 | 0.4 |
| 4 | 0.6 | 0 |
| 5 | 0.6 | 0.6 |
| 6 | 0.6 | 0.4 |
| 7 | 0 | 0.4 |
| 8 | 0.2 | 0.2 |
| 9 | 0.4 | 0.4 |
| 10 | 0.4 | 0.4 |
| 11 | 1 | 0.6 |
| 12 | 0.8 | 0.6 |
| 13 | 0.2 | 0.6 |
| 14 | 0.6 | 0.6 |
| 15 | 0 | 0.6 |
| 16 | 1 | 0.8 |
| 17 | 1 | 0.8 |
| 18 | 1 | 1 |
| 19 | 1 | 1 |
| 20 | 0.8 | 0.8 |
